# Distributed agency in HRI—an exploratory study of a narrative robot design

**DOI:** 10.3389/frobt.2024.1253466

**Published:** 2024-02-28

**Authors:** Philipp Graf, Christian Sønderskov Zarp-Falden, Lakshadeep Naik, Kevin Bruno Lefeuvre, Emanuela Marchetti, Eva Hornecker, Mads Bergholdt Sørensen, Laurits Valberg Hemmingsen, Ebbe Vincent Just Christensen, Leon Bodenhagen, Norbert Krüger, Andreas Bischof

**Affiliations:** ^1^ Media Informatics, Faculty of Computer Science, Technische Universität Chemnitz, Chemnitz, Germany; ^2^ The Maersk Mc-Kinney Moller Institute, Faculty of Engineering, University of Southern Denmark, Odense, Denmark; ^3^ Human Computer Interaction, Faculty of Media, Bauhaus-Universität Weimar, Weimar, Germany; ^4^ The Department of Media, Design, Education and Cognition, Faculty of Humanities, University of Southern Denmark, Odense, Denmark; ^5^ Danish Institute for Advanced Study (DIAS), University of Southern Denmark, Odense, Denmark

**Keywords:** human-robot interaction, distributed agency, robot design, interaction design, qualitative study

## Abstract

We explore an alternative approach to the design of robots that deviates from the common envisionment of having one unified agent. What if robots are depicted as an agentic ensemble where agency is distributed over different components? In the project presented here, we investigate the potential contributions of this approach to creating entertaining and joyful human-robot interaction (HRI), which also remains comprehensible to human observers. We built a service robot—which takes care of plants as a Plant-Watering Robot (PWR)—that appears as a small ship controlled by a robotic captain accompanied by kinetic elements. The goal of this narrative design, which utilizes a distributed agency approach, is to make the robot entertaining to watch and foster its acceptance. We discuss the robot’s design rationale and present observations from an exploratory study in two contrastive settings, on a university campus and in a care home for people with dementia, using a qualitative video-based approach for analysis. Our observations indicate that such a design has potential regarding the attraction, acceptance, and joyfulness it can evoke. We discuss aspects of this design approach regarding the field of elderly care, limitations of our study, and identify potential fields of use and further scopes for studies.

## 1 Introduction

Against the backdrop of the need for increased efficiency, work safety or even demographic change in societies of the global north, the use of so-called service robots is often presented as the solution, or at least an important part of the solution [see for the realm of care robotics (Lipp, 2019)]. Thus, the scenario of robotic agents that are able to work with, act on and react to human interaction partners in a multi-functional way—a concept that key figures in the field of robotics, including (Engelberger 1989), championed already in the 90 s—dominated the imagination and design approaches of robotic projects so far. At the same time, most robots in these scenarios share the characteristic of being conceptualized as a unified agent with a single coherent body. Prominent examples of this concept are the robot Sophia from Hanson Robotics (Riccio, 2021), Pepper from Aldebaran or the Care-O-Bot from Fraunhofer IPA (Graf et al., 2009). Yet this does not reflect the underlying technology and its capabilities. Robotic systems actually consist of an assemblage of components with distributed “intelligence.”

Additionally, studies and experience with universal and unified embodied robots show that often these scenarios are neither technically feasible in the near future nor socially wished for—see for the field of care (Van Aerschot and Parviainen, 2020; Maibaum et al., 2022). Nevertheless, most relevant stakeholders in the field of elderly care like workers, managers and insurances agree that there is a potential for specialized service robots bringing relieve from repetitive or strenuous work. These service robots may not necessarily be supposed to directly interact with humans, but nevertheless share the same space or cooperate with them from time to time. These robots should be designed in a way that matches their competences and limitations (Goetz et al., 2003; Walters et al., 2007), thereby affording a fitting mental model of the robot that makes their action and behavior understandable and—at best—also predictable for a human observer.

The work presented inquires into how we might conceive a robotic design that more accurately represents the assemblage-like nature of robotic agents, with the intent of fostering a deeper understanding of their functioning. We explore a robotic design that challenges the conventional concept of unified bodies by adopting a distributed agency approach, as introduced by Krummheuer et al. (2020). In this specific case, we aimed to examine whether this design approach can facilitate comprehension of the robot’s behavior and enhance the enjoyment of the human observers’ experience. We apply the notion of distributed agency to the design of a robot, where we implement different elements with several levels of agency within a single device. Concretely, we designed the base platform of the robot as a ship and placed a small agent on top of it, seemingly in control of the entire vehicle. In this way, we wanted to question the dominant design approach of robots as single-bodied agents and explore an alternative design that incorporates different—seemingly autonomous—elements that create a coherent narrative.

**FIGURE 1:**
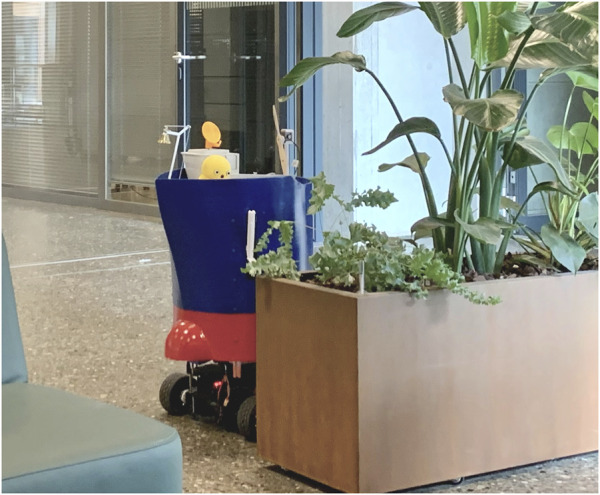
The PWR during the test on campus.

As we wanted to study a distributed agency design for robots in a field setting and to give it a meaningful position within its context, we needed a pretext task that our experimental robot could fulfill. We decided on the function of watering plants, as this is a regular-needed task that does not require direct interaction with humans. Based on this pretextual task, it was given the name Plant-Watering Robot, or PWR for short. Figure 1 shows the final robot prototype during the evaluation at one of the field sites, the campus. Through the construction of the PWR, our objective was to investigate the overall potential of a distributed agency design for robots functioning in social contexts. We hope to create an experience of a robot presence that is entertaining and joyful to watch, that stimulates social interaction, as well as the robot being “readable” regarding its behavior. In HRI, there is a strong focus on studying acceptability of robots through a questionnaire-based approach. We instead opted for a different approach, by observing people’s *in-situ* reactions and actual behavior with the robot. This aligns with recent discussions of a non-dyadic understanding of robots in social situations (Hornecker et al., 2022).

Because of the potential of robots acting in elderly care, one focus of our exploratory study was to investigate its functioning in a residential care home for people with dementia. Specifically, we aimed to evaluate whether this particular design can contribute to the needs of this particular user group, namely to promote the readability of specific and therefore functionally designed robots that can often cause discomfort when interacting with people with dementia (Grimme et al., 2021). Moreover, we wanted to explore how this robot could be something that is entertaining to watch, in order to lighten up the mood in this setting and to provide opportunities for social interactions between residents. Additionally, to gain a clearer understanding of how the design is perceived, we conducted field tests in the lobby of a university campus, which enabled us to inquire how the design is interpreted and described by younger users.

The paper is structured as followed: In the background Section 2 we reference the robot Cero that inspired our work, after talking about the relationship between robot bodies and their perceived identity. We draw on the literature of non-dyadic HRI to present the PWR as a concept that implements a non-dyadic interaction structure in itself. In Section 3, we elaborate on the design rationale in more detail and describe the final robot and its elements. In Section 4, we address the specific research interest arising from the design in more detail and present concrete questions that guided the study analysis. Section 5 presents our findings showing that the distributed agency design bears a potential to foster attractiveness of a service robot. Finally, we discuss the results, limitations of our study, and provide an outlook on further opportunities for studies related to distributed agency design.

The presented work was developed in the ReThiCare project, a research project that explored the design space of non-conventional (i.e. non-anthropomorphic, autonomous assistive tech) robotic concepts for care contexts. The interdisciplinary project brings experts of Human-Computer Interaction (HCI), sociology, robotics and product design together and grounded its work in an empirical field work phase in residential care homes at the start of the project. The goals were to develop new concepts that support and enable care work, while respecting its everyday routines and the values of dignity, autonomy and wellbeing. The specific aim for the PWR was to develop a new design and interaction concept that would enable robotic machines to work in socially complex situations, but would still be playful, poetic and appropriate to the context.

## 2 Background

The concept of the PWR relies on two strands of arguments. The argument made in Section 2.1 draws on the relationship between body and identity, while the argument made in Section 2.2 builds on the notion of non-dyadic interactions. Section 2.3 refers to Cero as a precursor of our idea.

### 2.1 Robotic bodies, identities and agency

In almost all cases, the design of robots is guided by the idea of reproducing delimitable and uniform actors (see Deng et al., 2019 or Kakoudaki, 2014) that possess agency. The automata of the eighteenth century already aimed at a “*reassemblage* of the whole” (Landes, 2007)—here still as a component of an epistemic practice that aimed to generate knowledge about the functioning of life through its simulation (Riskin, 2003). This is in line with the dominant perception of actors in the “natural” world when interacting with organic entities such as animals or other human beings (Plessner, 1975). In this context, Jackson et al. mention that a human’s mind, body and identity “are typically understood as having a default 1-1-1 mapping” that inherently connects each of those three aspects (Jackson et al., 2021). It can be questioned whether this mental model is appropriate when dealing with robotic agents, as those consist of different technical system with a varying degree of integration. Still, especially when relying on implicit modes of assessment, people tend to attribute “cognitive or emotional states” (Duffy, 2003) and related concepts, such as intentionality or morality (Zlotowski, 2015), to robots, thereby granting them high levels of agency.

However, from a social science perspective, agency is not something that is simply effectively present; instead, it is socially and communicatively attributed (Luhmann, 1984; Dickel, 2021)—this has been empirically proven also to be the case for interaction with robots (Alač et al., 2011) or androids (Straub, 2016). As Heider and Simmel (1944) could show in an early study on the ascription of agency to abstract shapes, the illusion of interacting with autonomous entities is primarily rooted in perceiving their movements as independent. Intriguingly, while the general comprehension of the narrative in the original study remained consistent in subsequent replications, the characterization of the geometric shapes involved became less anthropomorphic and more object-oriented over time (Lück, 2006). This shift could be interpreted as a growing acknowledgment of non-human agents possessing agency.

The ascription of agency is hereby oriented towards the boundary of the body—even if different body parts can be assigned to be “in charge” of concrete actions, the whole body is still seen as one unified actor that is “responsible.” This relationship changes significantly when dealing with assembled and interconnected technological objects, such as robots. From a social science perspective, we can describe this as a major shift in the societal perception of basic ontological categories of how entities are getting defined.

Regarding robots, this shift can (already) be measured empirically. For example, Williams et al. (2021) could show that a robot’s body and its identity are not perceived as strictly coupled. Instead, a differentiation between body and identity is applied, whereby the identity is seen as something that can be located in multiple bodies. Body and identity do not converge, but more complicated mental models emerge, where, for example, an artificial identity can switch between multiple robot bodies (Williams et al., 2021). Thus, in the case of robots and from a user perspective, the connection between body and cognition is perceived as variable and changeable. Or in the words of Jackson et al. “there are many well-known strategies for organizing robot minds, bodies and identities beyond simple 1-1-1 correspondence, especially in multi-robot systems composed of multiple robotic bodies, minds, and/or identities” (Jackson et al., 2021). It thus follows that the agency of a robotic agent also is no longer limited to a single body, but can be flexibly attributed to other bodies. This is also reflected in the development of different concepts like “one-for-all” system architectures (multiple bodies controlled by one entity), re-embodiment and co-embodiment (Luria et al., 2019). We complement these concepts with the notion of distributed agency (embodiment) design, highlighting the use of elements with different levels of agency within one robotic “body.”

### 2.2 Non-dyadic HRI

The notions of non-dyadic Hornecker et al. (2022) or triadic interaction (Krummheuer et al., 2020) concepts underlies the interaction design proposed with the PWR. The argument made is that a successful HRI depends on its social situatedness, which means that other human actors may be needed to sustain or facilitate an interaction—this seems to be especially true with sensitive user groups such as children (Alač, 2016) or the elderly (Chang and Šabanović, 2015), and as we will show in Section 5, is also true for the PWR, which often gets framed and explained by care staff. Hornecker et al. (2020) empirically showed that the use of technology in care work is always moderated verbally, manually and emotionally to ensure a safe and fluid human-machine interaction. Consequently, the same physical environment, same number of human users, and same context can lead to divergent requirements for a robot depending on whether it is used in a maternity ward or an oncology ward (Mutlu and Forlizzi, 2008). Central to our argument here is that the social role of a robot always results from its situatedness in a given social context, i.e., the social frame spanned by present humans. The social role in turn is crucial for the question of how a robot is evaluated and, in particular, whether it is perceived and accepted as a social actor or subject. Put simply, this evaluation has a continuum, whereby a robot appears as a social subject if it fulfills the role expectations directed at it (Meister and Schulz-Schaeffer, 2016) and more as an objectlike machine if it does not (Alač, 2016; Graf and Treusch, 2020). As discussed by Lipp (2023), it constitutes a special case when developers of such technology employ practices to ensure that robots can operate effectively outside of laboratory conditions.

But not only the presence of more than one human has an effect on the dynamics of HRI, but also the presence of more than one robot. Empirical results from Fraune et al. (2015b) show that the number of robotics agents interacting in a situation significantly changes how they are perceived by an observer—while effects of anthropomorphism decrease, stereotype attribution increases: zoomorphic robots appear more animal-like, mechanomorphic more machine-like and humanoids more human-like. Also, people tend to interact more with a group of robots than with a single one, and “reported them to be friendlier and more helpful,” as they get interpreted as being more social when interacting with each other Fraune et al. (2015a). While Smith et al. (2021) showed in a recent meta-review that not all effects of interaction with robot groups are well understood yet, it has been found that robot groups are rated more positively when they behave in a cooperative manner.

A robotic ensemble thus, through its design, contributes to how it becomes socially situated, by inviting specific interpretations and social interactions. While any robot, once embedded in a situation, will invite interpretations and interactions, it is the specifics of the design that can modulate what kind of interpretations are likely to be evoked and how social interactions unfold.

With the design rationale presented, we take advantage of the notion of non-dyadic interactions by implementing an additional non-dyadic narrative story within one larger object. By including several smaller elements with varying levels of agency within a larger robotic body, our objective is to achieve a multi-agent choreography that imparts its “own situatedness” within the wider social context. Consequently, users are encouraged to assume an observational role regarding the robot, rather than engaging in direct interaction with it. By displaying the story of a small robotic agent that is in charge of the overall behavior of the larger device, we hope to trigger similar effects as when interacting with a group of robots, that is, this ensemble being interpreted as friendlier and more helpful. To our knowledge, only one similar attempt has been made so far, namely the Cero robot.

### 2.3 Precursors from HRI

The interaction concept presented here has at least one important precursor in the field of HRI, namely the Cero robot built by Hüttenrauch and Severinson-Eklundh (Hüttenrauch, 2002; Severinson-Eklundh et al., 2003): Cero was a medium-sized service robot that supported an immobile user with fetch and carry tasks in the office. For us, the most important feature of the Cero robot was a small animated figurine (called character) on top of the robot that complemented the language and GUI interface and gave additional visual feedback on user requests or the overall state of the robot, for example, it waved its arms when the robot was moving. While the team that developed the Cero robot conducted several studies, none of them focused specifically on the animated character and the specific role it plays in its social context.

We build on the idea of using a small figurine that is in charge of the robot’s actions and visualizes the robot’s state of operation, but extended this approach in two main aspects: Firstly, we aligned the overall form design of the whole device to our little figurine in order to foster its control aspect. We, therefore, built the robot base as a ship, on which the little figurine appears as a captain next to a control panel. Secondly, to enhance the control illusion, we added additional elements on the ship’s deck, some of which additionally visualize the robots’ (upcoming) behavior, serve as interaction partner for the captain, or as simple communication device. On the one hand, we adapted the design of the robot so that it fits coherently into the distributed agency approach and, on the other, we extended this idea by adding further elements which are also intended to represent an agency aspect.

## 3 Design of the PWR

In the following, we first discuss the conceptual design concept of the PWR in Section 3.1, before we go into the individual elements of the concretely implemented design in Section 3.2.

### 3.1 Design rationale

As mentioned, the PWR was developed within the context of a project exploring alternative robot designs for the care sector. Early fieldwork in elderly care homes had highlighted the monotony of everyday life, especially for residents living with dementia, but also revealed instances of where this monotony was broken by social interactions, by visitors, or for instance a staff’s pet dog. The larger goal and context for the robot design was thus to make it entertaining to watch, so as to lighten up the mood, and to provide opportunities for social interactions.

The PWR explores a novel design rationale we call “distributed agency design.” This is a design that creates multiple and interacting affordances to interpret the robot not as a unified agent, but rather as an interplaying assemblage of several actors and connections—including the user’s actions and assumptions. It tackles the existing dominant design of robots as it takes account of the notion of agency ascription and situatedness laid out in Section 2. It does so by building the narrative of an internal control relationship between an agent that interacts with the rest of the device, namely the “ship” it sits on, and other elements—it thereby creates an additional situation “in itself” that adds to the social situation around it.

The goal of our design was to explore new ways to foster entertainment and acceptance of a robot. We aim to entice those present into an observer role, by offering an interesting to watch device, that entails sort of an interaction in itself, telling a narrative about its own functioning and purpose. The key element of this narrative is the representation of an agency or control relationship, which can be considered a well introduced cultural motive: a subject steers an object or animal. Even if this story may be deemed simple, it can be understood without language, referring to culturally well established motifs and role descriptions, such as the “helmsman,” “machine operator,” “shepherd” or “horse-rider.” For an observer, these motifs function as signs of the role or position within a depicted situation. Lefeuvre et al. (2021) refer to design approaches that offer a strong narrative to an observer as “robotic ensemble” or “robot as theater.”

**FIGURE 2:**
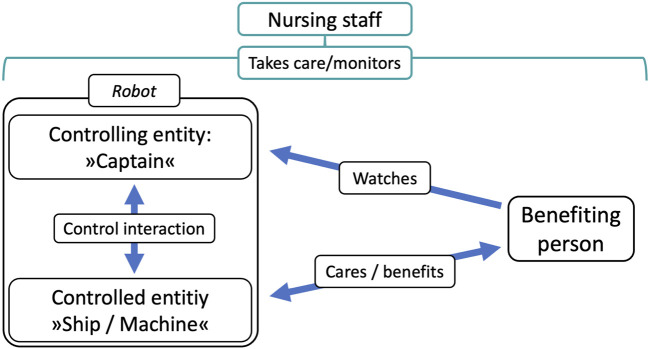
“Triadic” interaction concept of the PWR.

The box on the left side of Figure 2 depicts this ensemble, which is a control relationship between a controlled entity (in our example, “the ship/machine”) and a controlling entity (“the captain”). On the right side of the graph are the main addressees of the design, the care home residents that observe the robot’s behavior and benefit from its overall actions—in our case the plant watering activity. Above these are the nursing staff, which are in a supervisory position and monitor the overall situation.

**FIGURE 3:**
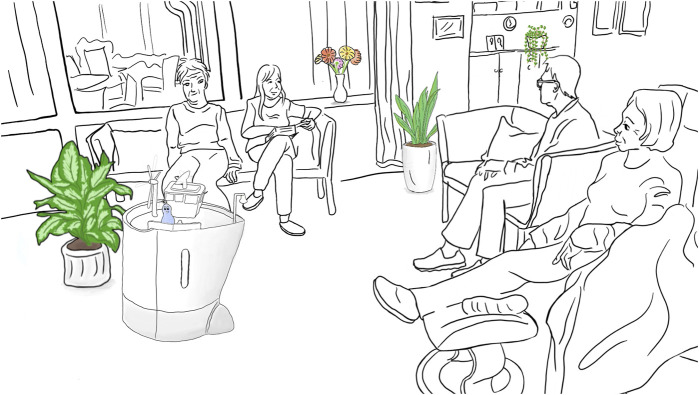
The Plant Watering Robot depicted in a common room of an elderly care home.

Figure 3, taken from a video prototype that served as discussion stimulus for a focus group, shows the PWR navigating the common room of a residential care home, being watched by a few people. We hope that the control relationship and the overarching theatricality of the robot will not only increase subjective acceptance and create delight, but also transform the robot into a conversation piece. A conversation piece is an object that, due to its design, stimulates social interaction by becoming a subject of conversation. This phenomenon has already been observed with existing robots deployed in care contexts, such as Paro (Hung et al., 2021). In the focus group interviews 3 with care professionals and informal carers, these had recommended extensions of the functionality, such as the ability to play music or to read out text aloud, as well as the ability to talk to the Keepon as a companion. Participants also pointed out that the robot should be big enough so it cannot be overlooked and become a tripping hazard, and that it should not cause extra work for care staff, i.e. it should be able to act autonomously. Whether and how the design would work, the participants said, could only be assessed empirically.

### 3.2 Description of the implemented design

**FIGURE 4:**
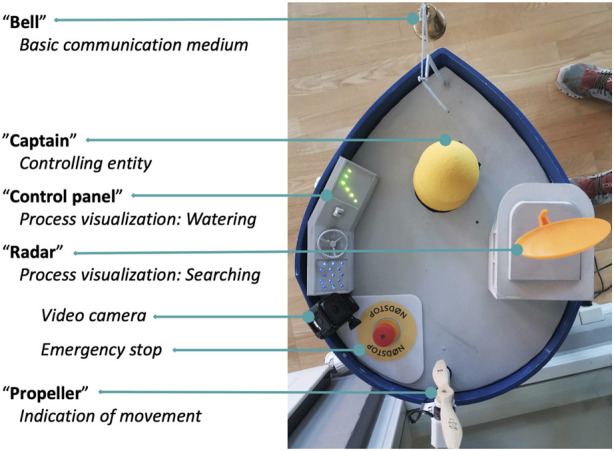
Overview of the deck elements of the PWR. Not visible in this birds-eye view is the retractable watering hose at the side of the ship.

In the following, we describe the final design implemented and explain the reasoning for concrete design decisions. We decided to design the PWR as “a ship steered by a small captain” because this motif is well introduced in cultural narratives and therefore widely understandable. It is part of both playful and classic stories since long time. The small captain embodies the controlling entity, which is supposed to draw attribution of agency to itself by showing autonomous movement—it can swivel around, wiggle from one side to the other, and jump. It sits on top and in the middle of a machine which is designed to resemble a deep sea vessel. To tailor this to the concrete context of elderly care, we chose a clear-cut and colorful form design, as well as conveying the control relationship very strongly within this context. The “ship” is signified through its form and bright colors. The role of the “captain” is represented by its position in the middle of the “deck.” To strengthen the control relation, but also the overall narrative, we added supplementary mechanical elements that fulfill different functions within the overall behavior during the task fulfillment of the robot. Figure 4 provides an overview of the deck of the ship. In the following, we shortly elaborate what elements it contains and how they work.

*The “bell”* is a one way communication medium and can draw attention to the robot by eliciting a high-pitched analog sound (e.g. when the robot enters a room). It consists of a metal bell that can be rung via a cord and a servo motor.

*The captain* is represented by a “MyKeepOn” (in the following referred to as “KeepOn”), a consumer version of the KeepOn robot built by Kozima et al. (2009) for triadic interaction scenarios with children with autism. It has been used successfully in other studies in single or group interaction contexts (Leite et al., 2015). It bears several advantages for our use scenario: The abstract shape does not elicit expectations of spoken interaction, and at the same time looks creature-like and friendly. It has a soft surface and a solid constructed skeleton, thus can be touched.

*The illuminated “control panel”* next to the KeepOn supports its role as captain. The LEDs built into the 3D-printed interface depict the status of the watering arm and water flow.

*The rotating “radar”* visualizes that the PWR is on the search for plant pots it can water, thereby indicating a general “work mode.” It consists of a 3D printed radar dish moved by a servo motor.

*The spinning propeller* is synchronized to the robots’ movement and is to inform observers about the ship’s movement. It consists of a wooden propeller normally used in airplane models, mounted on a pivoting mast. The propeller rotates according to the ship’s speed, and the mast is aligned in the opposite direction the ship takes.

*The video camera* mounted next to the control panel is not part of the main design, but added for user studies, and provides a close-up perspective for data analysis.

*The emergency button* stops all activities by the robot immediately, and is a standard safety measure.

Further, *a watering hose* extends from the side of the ship (not visible in the photo), which can extend forward and then spit water out, and then retracts.

To foster the impression of a relationship between those elements, forming a coherent narrative, all parts should behave as part of a choreography in which the captain accompanies every action of an element by turning its head towards it or to the control panel. The propeller indicates speed—moving accordingly in speed—and direction of the ship—by pointing into the opposite direction. A previous study with a preliminary prototype of the PWR had found that the approach of conveying the intended movement direction of the robot via such additional elements (Palinko et al., 2021) was understood well by viewers. The radar rotates when searching for plant pots. For the exploratory study, the overall choreography was remote controlled by two researchers. We consider this did not have a severe impact on our study, as we will elaborate further in Section 6. Another aspect of the narrative is the speed and behavior of movement of the whole “ship.” We moved the ship very slowly, ponderously, with only the front facing forward.

## 4 Research design

We first explain the study design in Section 4.1, before discussing the ethical considerations for the study in Section 4.2. In the last Section 4.3 we describe the questions that led the analysis of our qualitative analysis.

### 4.1 Study design

To evaluate our distributed agency robot design, we conducted a qualitative field study in two distinct settings, using a combination of settings for data collection. The settings were chosen for complementary insights. One study was conducted in a care home for people with dementia, collecting video-graphical data, focusing on behavioral data, while the other run was at a university campus, with the aim to collect explicit descriptions of the robot, giving insight into observers’ reasoning. Our use of video-graphic data and its analysis (Heath et al., 2010; Tuma et al., 2013) builds on research methodology developed in ethnography and sociology, which has been introduced into human-computer interaction research since the 2000 s, but is still rare in HRI. On the one hand, one can argue that relying on just observation only gives us indirect access to what people think and feel (other than interviews or questionnaires), but on the other hand, this allows us to see how people behave and react in a concrete (and not imagined) situation and social setting.

Despite the relatively small sample sizes (15 residents and 21 students), our study yielded valuable insights by amalgamating data from these distinct contexts. The study part at the university provided explicit descriptions of the robot from relatively young participants (mostly students). The student interviews were used to gain an initial understanding of how a healthy, able-bodied and above-average educated population perceived the design. This data has therefore been included in the analysis as a background foil, although it can only be evaluated in the specific context of the survey and the observer’s point of view. In contrast, the second setting involved observations of reactions towards the robot by elderly individuals in a care home, almost all of them living with various stages of dementia. This approach not only enabled us to examine two distinct participant groups, but also allowed for the integration of implicit behavioral patterns with explicit data concerning descriptions and evaluations of the robot.

#### 4.1.1 The care home setting

The robot was deployed in a care home for people with dementia on three subsequent days in the common rooms to simulate its anticipated use in an institutional environment. We planned to be for one hour on each floor in order not to overwhelm residents in dealing with the new device in their home environment. 15 different residents on two floors were recorded in 27 distinct situations, with one to maximum seven residents present. Based on societal gender cues, nine residents were identified as female and six residents as male individuals. In two situations, no reactions could be observed. So as to be able to make statements of whether a reaction has taken place or not, we define situation very broadly as an event in which the PWR is present in the area of perception of at least one resident. We ran the studies in the afternoon, as this is the time when residents are most active and responsive.

As study procedure, we let the robot move between two plants that got “watered” using a wizard-of-oz (WoZ) technique and tried to stick to the choreography mentioned in Section 3.2 as best as possible. As the robot should attract the attention of the residents while fulfilling its task, we did not approach them directly, so as not to evoke interpretations of intentional or attention seeking behavior.

Here, we could not conduct interviews with residents because they were in at severe stages of dementia. The management had emphasized that the attempt to interview them could overwhelm people with dementia, triggering emotions of stress and discomfort. Consequently, our qualitative investigation relied on a purely video-graphic analysis, a thorough and sequential analysis of audiovisual data from various perspectives aiming at people’s interpretations (Tuma et al., 2013). Additionally, we documented interpretations from caregivers concerning residents’ daily conditions, interactions or statements, where feasible. We conducted an in-depth analysis of the collected video data by sequentially reconstructing the patterns of interpretations of people observing or interacting with the PWR.

#### 4.1.2 The university setting

In a university cafeteria foyer over 2 days, data were collected in 15 interviews with overall 21 individuals passing by and observing the robot. Using the WoZ technique, the robot was driven around a hallway at the entrance to a café on campus. We approached those students who looked at the robot for a longer period of time or tried to interact with it and asked them for their consent for an interview. We then asked them, how they perceived the PWR and to verbally describe it. We chose this location, as the cafeteria lies between different faculties, so we could reduce the risk of having solely interview partners of the same disciplinary background. The interviews aimed to elicit a general description of the robot, to investigate the comprehensibility of the concept design. The interviews were analyzed with regard to the question of how the design of the robot was interpreted and which categories were used to describe the encounter with the PWR. The descriptions of the categories were used to draw conclusions about the attribution of agency or control relations (“who is in charge of the device”).

### 4.2 Ethical considerations

One site of study, the residential care home for people with severe dementia, bears ethical risks and practical challenges that must be considered when deploying a robot in a study (Ienca et al., 2016). The primary consideration is, of course, not to harm anyone or create feelings of fear or discomfort. The authors were diligent in adhering to ethical guidelines, as outlined in the ACM Code of Ethics and the British Sociological Association Statement of Ethical Practice. Measures were taken to ensure the wellbeing and privacy of participants, with staff and researchers being present throughout the study (and able to intervene if deemed necessary) and the robot being remotely controlled. Any close or physical contact was initiated solely by residents. As mentioned, we did not approach residents with questions, as this could create discomfort in people with dementia, who easily feel overwhelmed when having to take decisions.

The study procedure was agreed upon with the management of the care home and approved by the ethical board of University of Southern Denmark. Furthermore, the study procedure underwent review and approval by the University’s legal department. This encompassed considerations related to personality rights and privacy, in compliance with the EU General Data Protection Regulation (GDPR). Consent forms and the process of obtaining consent were scrutinized and approved. Consent to the study was provided by the legal guardians of care home residents. In addition, the care home residents were informed by the staff that they were taking part in a study. However, due to their dementia, it was uncertain whether they fully comprehended the information. The ethics committee had accepted this procedure, and their advice for such studies was that when people with dementia are shown the object of interest or introduced to a situation, a positive or neutral response to this object or situation should be interpreted as affirmation and consent, and a negative response should be accepted as withdrawal from the study. This process of “implicit consent” is deemed preferable because for people with dementia, it is stressful to make explicit decisions—such as saying yes or no to taking part in a study—and would overtax them. Therefore, we conducted the study with utmost care, to be able to withdraw the robot from a situation in case a resident would show signs of distress. Additionally, to protect privacy, comic-style drawings based on video stills are utilized in this submission to protect the identity of participants, given use of identifiable images was prohibited.

### 4.3 Methodological goals

The video-graphic analysis of data from the care home setting focuses primarily on the analysis of situations and emerging interactions of residents between each other and with the robot. We here define a situation as a socio-material configuration where those present can potentially observe the PWR and which is structured by social relationships and attitudes.

The video recordings were analyzed regarding their sequential structure, which means that we focused on the interrelations of experience, actions, interpretations and knowledge of persons with the material configurations and social dynamics of a specific situation. As a comparison foil, we use the interviews with students in the university setting that were asked to provide a description of the design and what purpose they assume the device to have.

The findings in Section 5 cover observations regarding a number of research questions concerning different aspects of the design. Of course, these questions can only be separated analytically, as they converge in practice. The questions that we explore in our study and that led analysis are as follows:

1. Because of the playfulness of the distributed agency design, we ask whether *the PWR is able to facilitate social interaction (as conversation piece).*
2. Because of the colorful and typical form design, we ask whether *the PWR is perceived as “a ship”.*
3. Because of the autonomous behavior and its integration into the whole device, we ask whether *the small agent is perceived as a distinct entity that is in charge.*
4. As we aligned the single elements to the task and an overarching nautical theme, we ask whether *the additional elements are perceived as part of a coherent narrative.*

## 5 Findings

In the following, we present our findings structured by the questions formulated under 4.3. Based on the video recordings, we were able to classify and count different types of engagement with the robot. We distinguished between gazing behaviour, physical references such as waving at or otherwise trying to attract the robot, touch interactions, and concrete interactions with the KeepOn. In total, there were 46 direct interactions with the robot, where several forms of reference could be used at the same time, which are only counted once here (e.g. touching implies looking). While the questions 1. (*conversation piece*, Section 5.1) and 2. (*perception as a ship*, Section 5.2) can be answered positively, this cannot be answered conclusively for 3. (*KeepOn as captain*, Section 5.3) (here there is positive evidence from the interviews in the University setting, but for the care home study setting, there is neither positive nor negative evidence) and 4. (*coherent narrative*, Section 5.4). Section 5.2 and Section 5.4 refer specifically to the distinctly new aspects of our implementation of the distributed agency approach mentioned in Section 3.1, namely the creation of a more coherent narrative and the addition of other elements. Vignettes of observed situations as well as footage from the video cameras are provided. Snapshots from the visual recordings are presented as overlay drawings for privacy reasons.

### 5.1 The PWR as a conversation piece

**FIGURE 5:**
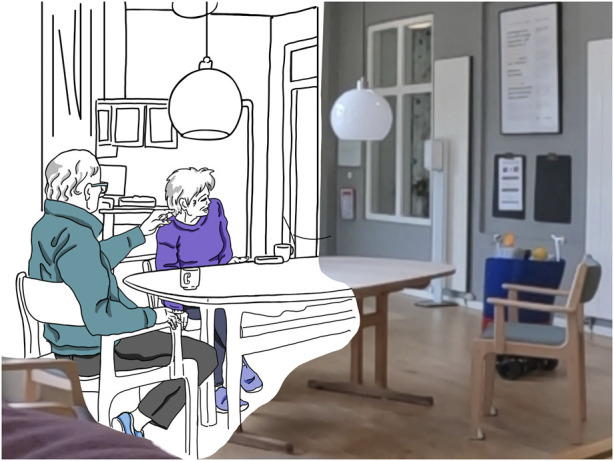
Male resident pointing to the PWR as it drives by and suggesting to another to look.

The question whether the PWR will facilitate social connections could be confirmed. In several situations in the care home, the appearance of the PWR led to residents starting to talk about it among each other, pointing to it cheerfully and asking others to look (16 distinct interactions between residents in total, see Figure 5), referencing the KeepOn and naming its function or functionality.

It also occurred that residents stood up during these conversations and took a closer look at the robot, or simply followed it with their gaze. This corresponds to the reactions observed during study part at the university, where the PWR aroused a lot of interest: Groups of students in particular, but also individuals, stopped to observe and talk about the robot—it is noteworthy here that the actual function of the robot could not be observed at all, but that the effect can be attributed solely to the narrative design. In this sense, the PWR constituted a successful conversation piece.

We observed an additional form of this function in the context of interaction with care staff of the care home. In some situations, the presence of the PWR was directly addressed by care staff and became the subject of conversation. This was the case when the PWR passed by a group of residents who were watching television with a member of the care staff, but also happened with individuals. In the latter case, the PWR was actively framed as a robot, but with varying degrees of success. In some cases it was ignored, in others it was spoken to.

The effect on people who came to visit the care home or on people who worked there, but were not interacting with residents, were interestingly low. In these cases, reactions were very spare, which we attribute to the fact that these people had a very strong goal or purpose at that moment—for example, to visit their relatives or fulfill a task. This highlights the significance of the observer’s situational role for perceiving the robot. It also emphasises that a narrative design is best suited for contexts with low stress and attention levels.

It should be noted at this point that these results must be evaluated cautiously against the background of the well-known novelty effect, because a new device in such a context of course is always exciting and worth talking about, simply because it is new. In three situations, the robot became not only the subject of a dialogue, but was also aggressively grasped and some residents tried to tip it over. We will come back to both aspects in Section 6.

### 5.2 The perception of the “ship”

The question, whether the design of the PWR was recognized as a “ship” or not, is difficult to answer in the context of the care home, since this interpretation is likely to evoke only a few specific physical reaction possibilities. We nevertheless found evidence that this was the case in some situations, as the following vignette shows:

On the third day of tests, we are on the second floor of the care home—eight people are present, spread across two tables, arranged in two parts of the open-plan common room. At one table, two caregivers engage with two residents, while at the other table, four residents have just taken their seats—it is thus relatively crowded in the room. We move the PWR along the wall towards the table with the four residents. The two people sitting closest to the robot, a woman and a man, take notice of the robot as it approaches. The woman stretches her hand out towards the robot—not wanting to grab it, but rather to attract it, as if she were interacting with a cat or other pet. The PWR does not react and continues to move along the table. As the PWR passes the man, he also extends his hand, but by doing so blocks the PWR’s path—the robot comes to a stop. Shortly afterward, he pulls his hand back again and the robot continues on its way. After about another meter, the PWR turns around on the spot and drives back in the opposite direction—this is also carefully observed by both residents. Shortly after, the man says to the woman, “it’s always moving with the front forward like a ship” [own translation].

Two things are noteworthy about this sequence: First, the different approach of the two people. While the woman addresses the robot as a perceptive and intentional being by trying to lure it, the man, blocking it, appears to want to test the device—he thus clearly interprets it as a technical object. Secondly, it is noticeable that he does not make his association of the robot as a ship dependent on the shape design, but rather on its movement behavior, i.e., the slow and only forward-directed way of driving—an aspect that we had considered intuitively, but did not assume to be dominant. At the same time, however, there is an implicit attribution in the man’s statement that the robot has a front and a back when referring to the “front” (of the ship), which also shows that the interpretation was not made independently of the form.

In another situation, the PWR leaves a room where a group of residents sits on a couch. One of them comments on its slow movement: “we should push it, so it moves.” Implicit in this statement is the assumption that the PWR is a vehicle that can be directed.

The results from the interviews with the students are clearer: when asked what the robot looks like, most of the interviewees expressed the association of a ship. Interestingly, this changed a bit when we asked them how they would describe the robot. Then, the focus was not so much on the aesthetic shape of the entire device, but more on naming the individual elements. We conclude that while the basic shape and color scheme worked well, the individual elements did not seem coherent enough in order to support the motif of a ship.

In summary, even if there are indications that the PWR was interpreted as a ship or at least as a vehicle, it cannot be said that our design was understood conclusively. Positively speaking, however, it can also be stated that the design did not trigger any major confusion or even fears at any time. We will come back to this in Section 6.

### 5.3 The captain as distinct entity that is in charge

With regard to the third question on the agentic character of the small captain, we could gather clear evidence in both observations and through the interviews, showing that the agent is perceived as a distinct entity from the ship. For example, there were at least three situations in which residents at the care home waved at the PWR with their hand as if to greet it while the KeepOn was moving. We interpret the waving as a reaction to the movement of the KeepOn and thus directed at the captain and not at the robot itself.

Interestingly, in two cases, this even happened with people at very advanced stages of dementia. From this, we conclude that the design of the KeepOn is very suitable for the context of elderly care to establish connectivity and social responses. In two other cases, people started to talk to the KeepOn. While these actions seem rare, they do show that the robot is perceived as an entity. Based on the gazing behavior and the focus of the residents, we interpreted the behavior as addressed towards the captain and not the ship as such. In one case the resident approached the KeepOn very closely (without touching it) and in the other case the reaction followed the KeepOn’s activity.

**FIGURE 6:**
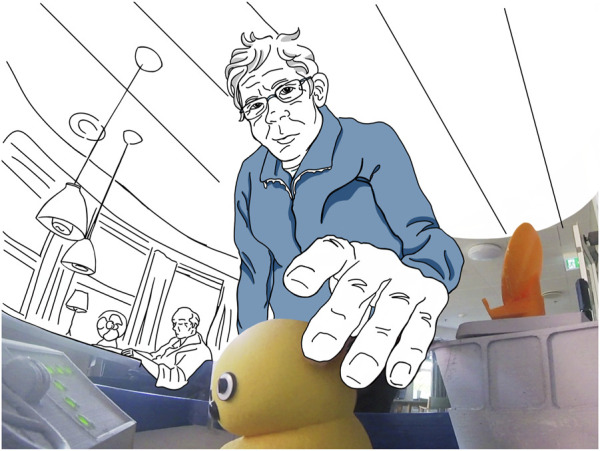
A resident pets the KeepOn as he walks by the PWR.

A clearer situation is captured in Figure 6, where a resident can be seen patting the little captain affectionately as he walks by, at the same time trying to figure out what kind of material it is. In another situation, where the PWR drives by a group of residents, one woman stated that “the duck on top is cute,” explicitly and positively referring to the captain as a distinct entity. These observations are backed up by description from interviews with students, who almost all differentiate the KeepOn from the rest of the robot, often referring to it as “cute.”

While all this shows that in the context of the care home, the captain was perceived as autonomous and as a distinct entity on the ship, we could not find clear evidence that it was also perceived as the centrally controlling entity (but also did not find evidence for the contrary). This may be due to the fact that this requires a more complex interpretation or that we did not collect enough data. Also, it might be difficult to clearly infer this from behavior alone. In the context of the university setting, we actually did find evidence of people perceiving a control relation. When asking students how they would describe the PWR, they said that “the duck does know what is going on,” “a robot that is controlled by that little duck” or they referred to the KeepOn as “little pilot.” All these descriptions clearly implicate the agents’ role as “being in charge.”

### 5.4 The additional elements as part of a coherent narrative

The fourth question, regarding the function of the additional kinetic elements for the overarching narrative, also remains inconclusive. A positive effect was identifiable; for instance, nearly all participants who mentioned the elements in their interviews also characterized them with adjectives like “intriguing.” However, there was no clear indication that the elements contributed to stronger coherence of the general narrative. In the following, we briefly focus on the four elements bell, control panel, radar, and propeller and describe reactions and descriptions to them.

The bell—to our surprise—generated significantly less attention than expected. While it was clearly noticed by bypassing students at the university, and also mentioned in interviews, we could not observe any reactions in the context of the care home that could be specifically attributed to the bell.

The control panel, on the other hand, was touched by care home residents in at least two cases, although it is unclear whether they recognized it as such. Statements from the interviews, where the device was described as a “robot that is controlled by a little rubber duck,” indicate that it could fulfill its specific function of supporting the role of the captain—but that might have been too complicated for the care home context. Based on interviews with caregivers, we attribute the reaction of touching the control panel to the fact that it is illuminated, as this often leads people with dementia to touch an object.

There were only few clear reactions to the radar in the context of the care home. Although it was identified and referred to by name in the interviews, there were no explicit statements or reactions to it from the residents. Nevertheless, its constant turning movement may have contributed to the liveliness of the entire robot.

**FIGURE 7:**
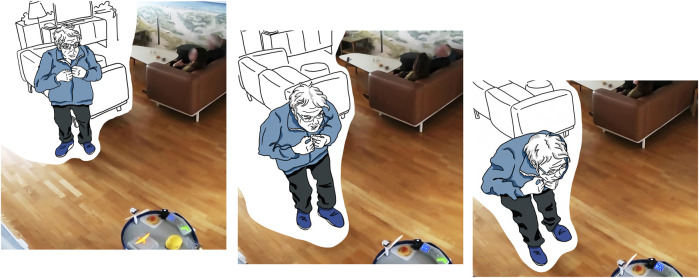
An elderly person approaches the robot from behind and blows on the propeller attached to the back of the robot.

The most interesting observations concerned the propeller, because this created the strongest reactions. While it was associated with “green energy” by the students, the reactions of care home residents were more varied and playful. In one case, when the PWR drove past a group of residents and a conversation about it ensued, they speculated that the propeller could fan them with wind when it was hot in the summer. It is noteworthy that the originally intended meaning of the propeller as a kind of propulsion was not understood, but that the propeller was read as a component with an additional function. In contrast, the following vignette, also shown in Figure 7, shows a rather playful interaction with the propeller, that supports our intended interpretation:

The PWR was close-by and observed by a group of residents sitting on the couch watching TV. Then, the PWR was about to leave the situation. It was supposed to drive back towards the larger common room. Shortly before, there was the aforementioned conversation about whether the robot should be pushed, so it would move faster. The robot’s leaving and its slow speed were also observed by an elderly man, who then decided to interact with it. He approached the PWR from behind, bent down and blew on its propeller, as if it were a sailing ship, and he wanted to move it forward.

Two things are noticeable here. Firstly, the man seems to have recognized that the propeller has something to do with the propulsion—he tries to support the propeller moving faster, and secondly, he interprets the situation playfully, as is often characteristic of people with dementia.

In summary, the elements thus had an influence on the perception of the robot, but this was much looser and more difficult to determine than we had hoped for. We will discuss possible reasons for this in the next section.

## 6 Discussion

We acknowledge several limitations in our study. The robot presented in this research and the findings from our preliminary investigation can only provide initial insights into the topic of distributed agency design for robots. The limited sample size, the absence of care home resident interviews, the limited duration that we could expose the residents and students to the PWR, and the fact that we could only evaluate one specific design implementation of the overarching rationale prevent us from formulating definitive evidence or performance measures. However, we contend that our qualitative study design has allowed us to demonstrate the potential of the overall concept. In the following sections, we discuss our findings, focusing on the context of elderly care. Additionally, we delve into further research questions and potential social contexts for future investigation.

The results, as previously mentioned, are ambiguous. Despite the narrative and associated choreography not achieving the full intended outcome, it is evident that certain elements did indeed have an effect. One obstacle may be that the technical nature of the narrative and implemented design led to reduced comprehension, as indicated by interview statements and observed responses. Nonetheless, we discovered evidence suggesting that distributing agency to an additional agent (in our case, a captain) is a design choice that is comprehended by many, even though its integration within a narrative remains largely presuppositional.

The metaphor of the control relation demonstrated greater reliability in an environment populated by younger individuals with an affinity for technology, including students on a campus, compared to the care home setting. However, other aspects, such as the colorful and playful robot design, appeared to be effective in engaging individuals with dementia. It became evident that the distinction between robot and captain could elicit a significant response or attract attention, while the additional elements were less likely to do so. If any effects were observed, they were primarily attributed to visible movement of these elements, which made them more noticeable. This finding aligns with perceptual psychology research indicating that motion design tends to have a strong impact on capturing attention (Pratt et al., 2010). We postulate that a more cohesive integration within the overall design narrative and better synchronization among all elements would be necessary to enhance their role in the assemblage.

Despite these drawbacks, we were able to show that the role of the PWR as a conversation piece could bring a welcome and well-functioning distraction to the daily routine of the care home. We therefore conclude that the distributed agency approach might support the emergence of triadic interactional relations. Adding another elements to a robotic device allows for the potential to alter routines, keeping the robot engaging and captivating for an extended duration.

Overall, our observations also confirm a well known fact, namely that different people react differently to a robot. Some show interest, others none at all. On a positive note, however, the robot did not frighten any of the residents, as can be the case with abstract designed vacuum cleaner robots (cf. Grimme et al., 2021). It thus seems that the design has a trustworthy form, which leads to neutral to positive reactions. We suspect this is mainly due to the bright color scheme, the very slow speed and the generally friendly design of the KeepOn. To our surprise, there was almost no reaction to the robot’s noises, i.e. its own motors as well as the bell (possibly because many elderly people are hard of hearing). The technical nature of the object appealed more to male residents, as can be seen from the fact that it was mainly men who pointed at the robot and made it the subject of conversation. We also observed that those residents who had a technical profession showed a stronger interest in the robot.

The challenges encountered, including interactions with individuals with dementia who may act erratically or unpredictably when faced with stimuli, attempting to grasp or tip over the robot, highlight the inherent difficulties associated with constructing a robot that serves as an attraction in and of itself. If a robot is to succeed over time in this demanding environment, it would need to possess exceptional robustness and stability to avoid toppling over. Consequently, deploying these robots in areas where there is a higher appreciation for the delicacy of technical devices is more feasible. Moreover, the design rationale’s potential to incorporate intricate and elaborate narratives that enable behavioral variability suggests that this design approach would be well-suited for contexts involving long-term interactions between robots and the same users, for example within corporate environments.

Alongside previous work on design recommendations for robots used in elderly care (Bradwell et al., 2021), our work demonstrates the need for alternative design approaches that do not resemble stereotypical “robotic” aesthetics. The distributed agency design approach goes beyond this and promotes a playful and joyful character. Further research should address whether this design approach can effectively promote long-term acceptance of a robot. Additionally, investigating how diverse user groups respond to a distributed agency design in various contexts would be an intriguing avenue to explore, as it can contribute to the understanding of robotic design, particularly regarding the distribution of agency. An unaddressed question pertains to how this design may influence the wider interaction design with users. For instance, examining the potential effects of allowing users to engage in conversation with the captain or if the captain can directly respond to users when the robotic device is perturbed would be worthwhile to investigate.

## 7 Conclusion

We have elaborated the principle and possibility of a distributed agency approach for robots, which we argue to be more adequate for HRI than a 1-body design, to strengthen readability and lead to better accepted robots. We reported on the implementation of one such design, the Plant-Watering Robot, which is designed as a deep sea vessel that is controlled by a KeepOn robot acting as a tiny robotic captain. We presented a qualitative exploratory study conducted at both a university and a residential care home setting. The distributed agency design is based on the idea of creating a coherent narrative within a robotic device, that acts as if it is controlled by a distinct robotic entity. By employing a triadic design, we add a layer of interaction to the HRI that takes effect before any actual explicit interaction between humans and robots occurs: as an observable agency within the robotic system. The goal of this design approach is threefold: To take the pressure off people to interact with an autonomous robot, to make the device more comprehensible and acceptable by offering a coherent narrative that is consistent with the distributed nature of robotic objects, and—last but not least—to make the device more joyful and pleasant to watch when operating.

Our exploratory study could demonstrate the potential of an additional agent that appears to be responsible for the actions of the device, as it could attract attention and facilitate social interaction between residents of a residential care home. At the same time, the more elaborate maritime design narrative of the PWR was difficult for observers to access, leading to ambivalent results. Since the individual physical elements of the design elicited responses from some observers, we conclude that a simpler overall narrative for the triadic design would result in eliciting a more robust interpretation of the robot and the possible interactions it seeks to represent.

## Data Availability

The original contributions presented in the study are included in the article/supplementary material, further inquiries can be directed to the corresponding author.
